# Supplementary material for: A Literature Review on the Use of Aortic Allografts in Modern Cardiac Surgery for the Treatment of Infective Endocarditis: Is There Clear Evidence or Is It Merely a Perception?
Source: Life (Basel). 2023 Sep 28;13(10):1980. doi: 10.3390/life13101980 (PMC10608498; doi:10.3390/life13101980)
Supplement: Supplementary file 1 [file life-13-01980-s001.zip › life-2574035-supplementary.pdf]

Table S1 Meta-Analysis and Observational Studies Comparing the Aortic Allograft with Conventional Prosthesis					
**First Author or StudyAcronym (Ref. #)**	**Total Sample (N)**	**Number of Patients/ Endocarditis**	**Mean Follow-Up/Months**	**Number of Aortic Valve Substitute Implanted**	**Main Findings**
Nappi 2018 JTCVS (1)	210	118	162	AA (210) χAMA(11)	†Similar survival at 15yrs compared to stented xenograft 61.3% vs 62.1% and mechanical prosthesis 61,3% vs. 60,6% Reoperation SVD-related freedom from event at 15 yrs 89.4. Low indicence of reoperation for infection relapse. Freedom from IE 98.1% at 20 yr. MACCEs freedom from event at 15 yrs 50.6%
¥Wang 2017 Annals	42.305	Not in the inclusion criteria	Mean times to valve failure (MTTF)	Medtronic Porcine (9.619) Edwards Porcine (3.886) Sorin Pericardial (6.632) Edwards Pericardial (22.177)	Sorin pericardial showed higher SVD risk; p < 0.001 for all other three valve type (lower risk-adjusted MTTF). No significant differences in SVD risk among the other three valve types ((p= 0.716).
Kim 2016 JTCVS	#304	304	29.4	AA (86) Mechanical (79) Xenograft (139)	Similar survival between valve substitute. Overall death 19.7% (P = .23). AA did not significantly affect early death. Odds ratio 1.61; 95% confidence interval [CI], 0.73-3.40, P = .23 (HR 1.10; 95% CI, 0.62-1.94, P = .75). Reinfection 7.7%. No differernce in freedom from reinfection rates (P=.65). AA did not significantly affect reinfection (HR 1.04; 95% CI, 0.49-2.18, P = .93)
*Kim 2016 JTCVS	#436	IVDU 78 Non-IVDU 358	29.4	AA (86) Mechanical (99) Xenograft (206)	Similar survival between group (IVDU vs Non IDVU). ([HR], 0.78; 95% CI, 0.44-1.37). No difference between valve substitute. Lower operative mortality in IVDUs (odds ratio, 0.25; 95% confidence interval [CI], 0.06-0.71). Better valve-related complications in IVDUs (HR, 3.82; 95% CI, 1.95-7.49; P<.001) for higher rates of reinfection (HR, 6.20; 95%CI, 2.56-15.00; P<.001).
Perrotta 2016 Annals	84	84	65	AA (56) Mechanical (20) Xenograft (12)	Similar survival at 10 yrs. AA 58%vs conventionalprosthetis75% (P=0.17). Not significantly different at 30-day mortality AA vs conventional prosthesis (10.7% vs 9.7%; P = 0.88). Higher indicence of reoperation for infection relapse in mechanical or xenograft valve prostheses (12.9%) than AA (0%) (P= 0.006). Lower indicence of reoperation for SVD in AA at 10 yrs (5,3%)
Arabkhani 2016 JTCVS	353	115	137	AA (353)	Survival 40.0% at 20 yrs (95% CI, 32%-50%). 20 yrs predicted competing-risks analysis 31% death without reoperation, 39% reoperation, and 30% alive without reoperation. Low incidence of infection relaps (3,96 %) and reoperation (2,26 %)
Φ Foroutan 2016 BMJ	53 884	Not in the inclusion criteria	Cumulative incidence of Death and SVD at10,15 and 20 yrs	Xenograft (53 884)	Survival 89.7%,78.4%, 57.0%, 39.7% and 24.7% at 2,5,10,15, at 20 yrs. Freedom from SVD 94.0%, 81.7%, 52% at 10,15, and 20 yrs (evaluated for 7603 pts). The rate of SVD increases rapidly after 10 yrs, and particularly after 15 yrs.
Flameng 2015 Annals	69	69	96	AA (69)	Survival 73% at 10 yrs. Freedom of reoperation 74% at 10 years. Lower incidence of infection relapse and reoperation for IE (4,34%.). Higher indicence of reoperation for SVD (18,84%)
Bourguignon 2015 Annals	2 559	111	79	Xenograft (2 559) (Perimount pericardial bioprostheses)	Survival (including early deaths) 31.1%,14.4% at 15 and 20 yrs (95% CI). IE early 0.11%; late 0.38%/ [95% CI 0.30%–0.48%]. Freedom from SVD 94.2%, 48.5% at 10 and 20 yrs. MST 19.7years (95% CI 18.5% to 21.1%). Freedom from reoperation (60 to 70 yrs) for SVD 82.7% at 15 yrs and 59.6% at 20 yrs. Cumulative risk of reoperation for SVD HR 0.93 (95% CI 0.92 to 0.94; p <0.001)
Bourguignon 2015 Annals	373	39	102	Xenograft (373) (Perimount pericardial bioprostheses)	Survival 78.1%, 65.6%, 46.8% at 10, 15, and 20 yrs (95% CI). IE early 0.26%; late 0.52%/ [95% CI 0.31%–0.81%]. Freedom from IE 95.5%, 92.2%, 88.8% at 10, 15, and 20 yrs. Freedom from reoperation for SVD 88.3%, 70.8%, 38.1% at 10, 15 and 20 yrs. Competing risk analysis for SVD 41.6% at 20 yrs with (17.6 years of expected valve durability).
Fukushima 2014 JTCVS	840	101	420	AA (840)	Survival at 35 yrs 66%. Reoperation rate for SVD at 35 yrs 33,9%. Under continued follow up at 35 yrs 31, 7%.AA durability more than 15 yrs, 2 pts with AA for more than 30 yrs. Lower incidence of infection relapse and reoperation for IE. Early reinfection 0,2%. Late relapse of IE 5,5% (25% of response to antibiotic treatment).
Sénage 2014 Circulation	617	Not in the inclusion criteria	44	Xenograft (617) Mitroflow (models 12A/LX)	Survival 69.6% at 5 yrs (95% CI, 65.7–73.9). Early SVD of the Mitroflow (models 12A/LX). The 1-, 2-, and 5-year cumulative probability values of SVD were 0.2% (95% confidence interval [CI], 0.0–0.6), 0.8% (95% CI, 0.0–1.6), and 8.4% (95% CI, 5.3–11.3). 5-year SVD-free survival 91.6% (95% confidence interval [CI], 88.7–94.7). Related to valve size 79.8% (95% CI,71.2–89.4) and 94.0% (95% CI, 90.3–97.8) for patients who received 19- and 21-mm sizes. Among 39 cases of SVD, 13 patients had an accelerated SVD.
Amabile 2014 JTCVS	500	10	104,8	Stentless xenograft bioprostheses (500) Freestyle bioprosthesis (Medtronic Inc, Minneapolis, Minn)	††Survival 68.2% at 10 yrs. Freedom from SVD at 10 years 94% at 10 yrs (0.6% per patient/yr.). Freedom from endocarditis 96% at 10 years (0.5% per patient/year). Survival 83% in aged less than 65 yrs and freedom from SVD 89% at 10 yrs.
Garrido-Olivares 2011 Annals	#1076	12	166	Hancock II Bioprosthetis 1076	††Survival 57.5%, 34.1% and 19%, at 10,15 and 20 yrs. Reoperation on the AV for any causes 93.5%, 83.6% and 62% at 10,15 and 20 yrs. Reoperation for SVD 96.4%, 84.6% and 68,5% at 10,15 and 20 yrs.
Musci 2010 JTCVS	1136	1136	62	AA (221)	Survival at 30 days, 1,5,10 yrs 83.8% ± 3.7%, 76.6% ±4.3%, 66.5%±4.9%, and 47.3%±5.6%. Lower incidence (5,4%) of infection relapse and reoperation for IE. Lower incidence of reoperation for SVD 8,6%
David 2008 JTCVS	357	7	91	SPV (T-SPV) bioprosthesis (St Jude Medical, Inc, St Paul, Minn) (357)	Survival 64% at 12 yrs. The freedom from SVD 69% at 12 yrs (52% for patients less than 65 years of age, and 85% for patients 65 yrs of age or older (P = .002). Higher incidence of infection relapse (10% of redo aortic valve replacement). The freedom from redo aortic valve replacement 69% at 12 yrs.
David 2007 JTCVS (	383	383	73	AA (18) Mechanical (214) Xenograft (133)	Survival 44% at15 yrs. Relapse of IE independent predictor of death (HR 2.2, 95% CI 1.2-3.9). Freedom from recurrent IE 86% for all patients at 15 yrs without difference between type of valve implanted. Freedom from reoperation 70% at 15 yrs.
Yankah 2002 EJCTS	816	816	60	AA (182)	Survival at 1 and 10 yrs 97% and 91%. Lower incidence of early (2.7%) and late (3.6%) infection relapse and reoperation for IE (P=0.0001). Freedom from reoperation for SVD 85 % at 10-13 yrs

O'Brien 2001<br>JHVD	1 022	92	300	AA (92)	Survival at 25 years of the total patients 19 +/- 7%. Early endocarditis occurred in two of the 1,022 patient cohort, and freedom from late infection (34 patients) at 20 yrs 89%. One-third of these patients were responsive to antibiotics for IE. Freedom from reoperation for SVD at 15 years 47%
Moon 2001<br>Annals	306	306	183	AA (20)<br>Mechanical (65)<br>Xenograft (221)	Similar long-term survival excluded operative death at 10 yrs (62% mechanical, 61% bioprosthetic, 58% AA; p > 0.50) and at 20 years (46%, 41%, 58%; p> 0.27. Lower risk of infection relapse without group difference. During the first 5 years 2.1% mechanical prosthesis, 2.3% stented xenograft, and 3.6% AA; P> 0.88. After 5 years 0.5% mechanical prosthesis, 1.1% stented xenograft and 3.1% AA; P >0.25. Freedom from reoperation for mechanical prosthesis 74,6 % at 10 and 15 yrs; xenograft prosthesis 56.6 %, 22.6% at 10 and15 yrs; p> 0.64.

Abbreviations; AA; aortic allograft; AMA, aortomitral allograft; CI, confidence interval; HR, hazard ratio; IDVU, intravenous drug abuser; IE, infective endocarditis; MACCE, major adverse cardiac cerebrovascular event; SVD, structural valve degeneration.

Propensity Score (discrimination C statistics and calibration Hosmer-Lemeshow statistics); #Propensity score with covariate adjustment (propensity-adjusted hazard ratio testing of partial (Schoenfeld) residuals). * IVDU= intravenous drug user. Of total N= 436 Valve repair was performed in N= 45; MST =median survival time; ††includes the two groups no-RRA and RRA replacement; Φ Meta-analysis based on Metaprop's DerSimonian and Laird random effects model and Freeman-Tukey double arcsine transformation; ¥ Meta-analysis and meta regression. 54 papers evidenced a total valve counts that were studied with Weibull distributions and the mean times to valve failure (MTTF).
